# Prolactin-secreting pituitary adenomas: male-specific differences in pathogenesis, clinical presentation and treatment

**DOI:** 10.3389/fendo.2024.1338345

**Published:** 2024-02-02

**Authors:** Lukasz Dzialach, Joanna Sobolewska, Zuzanna Zak, Wioleta Respondek, Przemysław Witek

**Affiliations:** ^1^ Department of Internal Medicine, Endocrinology and Diabetes, Medical University of Warsaw, Warsaw, Poland; ^2^ Department of Internal Medicine, Endocrinology and Diabetes, Mazovian Brodnowski Hospital, Warsaw, Poland

**Keywords:** dopamine agonists, men’s health, pituitary adenoma, pituitary neuroendocrine tumor, prolactin

## Abstract

Prolactinomas (PRLomas) constitute approximately half of all pituitary adenomas and approximately one-fifth of them are diagnosed in males. The clinical presentation of PRLomas results from direct prolactin (PRL) action, duration and severity of hyperprolactinemia, and tumor mass effect. Male PRLomas, compared to females, tend to be larger and more invasive, are associated with higher PRL concentration at diagnosis, present higher proliferative potential, are more frequently resistant to standard pharmacotherapy, and thus may require multimodal approach, including surgical resection, radiotherapy, and alternative medical agents. Therefore, the management of PRLomas in men is challenging in many cases. Additionally, hyperprolactinemia is associated with a significant negative impact on men’s health, including sexual function and fertility potential, bone health, cardiovascular and metabolic complications, leading to decreased quality of life. In this review, we highlight the differences in pathogenesis, clinical presentation and treatment of PRLomas concerning the male sex.

## 1 Introduction

Prolactinomas (PRLomas) are the most common type of functional pituitary adenomas (PAs) (1–3). Prolactin (PRL)-secreting PA that occur in men compared to those diagnosed in women tend to be characterized by larger average diameter (4, 5), more aggressive clinical course (4, 6–9), and treatment difficulties (10, 11). These features also favor a different clinical picture in each sex, as large-diameter tumors may cause symptoms secondary to the mass effect (12–17). The diverse clinical picture of male PRLomas, varies from asymptomatic (18), through a predominance of hypogonadotropic hypogonadism (HH) features (7, 9, 12, 13, 15, 17–23), to invasive tumors with extrasellar extension (16, 24). Women tend to reveal menstrual disorders, a more common reason for early medical advice (12). In men, the diagnosis may be delayed due to unawareness of symptoms, their shaming nature for patients, or the timing of reporting to the physician since their onset (12–14, 19, 25, 26). In addition to the differences mentioned above, PRLomas in men also affect cardiovascular and metabolic risk (12, 27–29), semen quality and fertility potential (13, 30–34), bone health (12, 35, 36), and increase in total body fat (29, 37, 38), making the role of hyperprolactinemia control essential in men’s health.

PRL-secreting PAs are an exception among other PAs, as pharmacotherapy is the first line of treatment (39, 40). However, due to more frequent resistance to dopamine agonists (DAs) therapy (10, 11), the larger tumor diameter (4, 5), and the need for radiation therapy (41–44) or surgical intervention, PRLoma management in men may become challenging. Reviewing the literature, there appears to be less extensive data on male PRLomas. This review aims to highlight the differences in pathogenesis, clinical presentation and treatment concerning the male sex, with particular emphasis on clinical practice.

## 2 Epidemiology

PRLomas are the most common type of PAs and account for about 53% (39.9-66.2%) of them, with a marked female predominance (1, 2, 45–47). They are relatively rare and constitute approximately 19% of all PAs in male patients (48). According to their diameter, PRL-secreting PAs are classified as micro- (<10 mm) or macroPRLomas (≥10 mm) (21). Considering the available data from the past few years, the average age at diagnosis is 35-50 years in men and 28-44 years in women (5–7, 13, 15, 47, 49), without significant distinctions in the age of diagnosis comparing micro- and macroPRLomas (15). The female-to-male ratio in incidence is estimated at 5:1 to 10:1 between the ages 20 and 50 (6, 50), and after the fifth decade of life, the frequency of PRL-secreting PAs between women and men becomes equal (50, 51). However, some epidemiological data show a clear predominance of men among patients diagnosed with PRLomas after the age of 50 (up to 88%) (25). In contrast to women, in whom the highest prevalence of PRLomas occurs at the reproductive age and in the third decade of life (25), men do not show a clear peak in incidence (26). Indeed, data show a steady increase in the incidence of PRL-secreting PAs throughout life in men (25) or persistence at about the same occurrence in all age groups (5, 20, 47, 48).

In males, PRLomas are notably larger than those in females and, at the time of the diagnosis, are likely to be macroadenomas with high serum PRL concentration (4, 5). MacroPRLomas constitute approximately 75-88% of male PRL-secreting PAs, while women are more commonly diagnosed with microPRLomas (80-84%) (6, 25, 52). The difference in PRLomas size is already noticeable in children; tumors in boys are typically larger and more invasive than in girls (70 and 38%, respectively) but they are generally more frequent in girls (53–57). Up to 25% of male PRLomas are represented by giant PRLomas (gPRLomas) (24, 31, 58, 59), defined as PRL-secreting PAs ≥ 4 cm in diameter, often associated with markedly high serum PRL levels and significant extrasellar extension (16, 24). The incidence of gPRLomas is estimated at 0.5 to 4.4% of all PAs (60, 61). Unlike PRLomas in general, gPRLomas are predominantly diagnosed in men with a male-to-female ratio of approximately 3-9:1 (16, 24, 59, 60). Iglesias et al. showed that in men, the prevalence of gPRLomas might be even higher than microPRLomas (15). The mean age at diagnosis of gPRLoma in men is 29-49 years, and the age distribution is similar to non-gPRLomas (16, 24, 59). All cases of gPRLomas in children < 15 years reported to date were in boys (49).

PRLomas are also the second most common type of pituitary carcinomas (PC), representing up to one-third of them and approximately 0.04% of all pituitary tumors (62–64), and show higher male prevalence (64, 65).

Most often, PRL-secreting PAs occur sporadically, but up to 5% of cases develop as a component of the familial genetic syndrome, such as multiple endocrine neoplasia type 1 (MEN-1), MEN-4, Carney complex, familial isolated pituitary adenomas (FIPA) and familial pheochromocytoma/paraganglioma/pituitary adenoma (3PA) syndrome (66, 67).

## 3 Pathogenesis

As mentioned above, PRLomas in men, in comparison to women, are usually larger, more invasive and show higher PRL concentration at the time of the diagnosis (5, 14). The reason for the predominance of more aggressive tumors in men is controversial and not fully understood. One of the proposed explanations of that phenomenon is the diagnostic delay in males due to less evident (and sometimes even shaming to some) clinical presentation than in females. In women, high PRL levels typically lead to amenorrhoea-galactorrhoea syndrome, which prompts the diagnosis; at the same time, men are usually diagnosed because of a tumor mass effect (visual disturbances, headaches, hypopituitarism) and less frequently due to decreased libido or erectile dysfunction (12–14, 50). Nevertheless, this is not likely a comprehensive answer, and these sex-specific clinical differences are rather linked to distinction in PRLomas behavior. Indeed, several studies have suggested that male PRLomas tend to have higher proliferation activity, more frequently present cellular atypia and are well-vascularized (5, 20, 68, 69). However, because the vast majority of PRLomas are treated pharmacologically, studies attempting to correlate these tumors’ clinical and histological data are limited.

**Figure 1:**
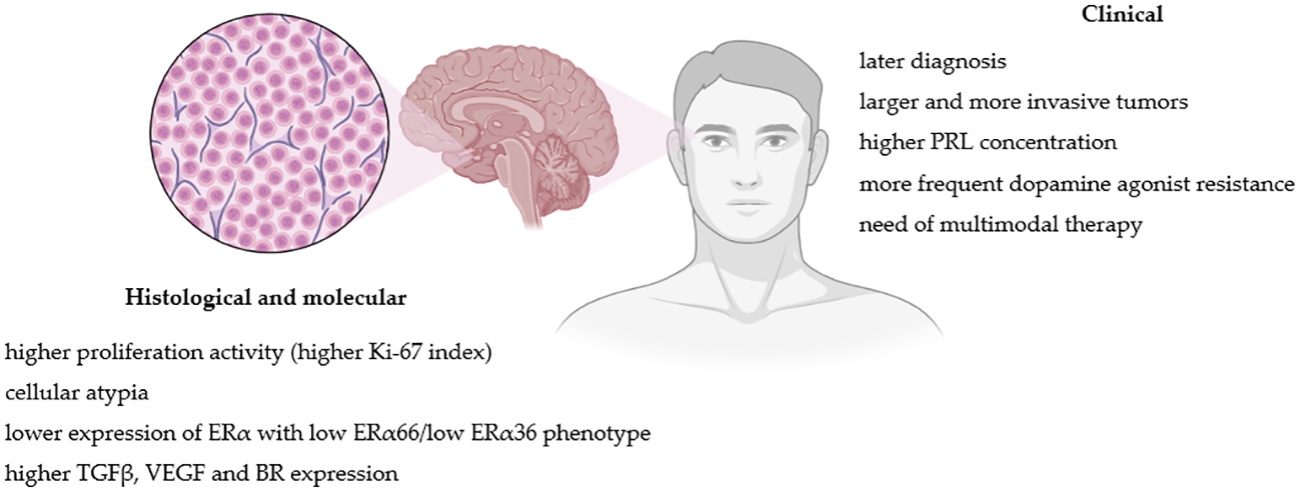
Pathogenic and clinical factors differences between lactotroph tumors in women and men. Figure created in BioRender.com.

Histological, molecular and clinical factors differences between lactotroph tumors in women and men are summarized in **Figure **1.

### 3.1 Proliferation markers

Most PRLomas present low proliferative activity determined by the Ki-67 proliferative index (17). It has been reported that higher Ki-67 correlates positively with tumor size, PRL levels and DA resistance; thus macroPRLomas and invasive tumors generally present significantly higher Ki-67 than microPRLomas (69, 70). Such observation has been made in both women and men (5). However, PRLomas in men present higher Ki-67 values than in females (5, 68, 71), also within the group with tumors of similar size (5). DA-resistant tumors, which occur mainly among men (10, 11), also present higher Ki-67 expression (5, 69). This greater proliferative potential observed in male PRLomas could explain the greater incidence of larger and more aggressive tumors in men.

### 3.2 Estrogen and progesterone signaling pathways

Several studies support the role of estrogens and their receptors (ERs) in PRLomas development and progression (68, 72–74). Normal lactotroph cells generally express ERs, as most PRLomas do (75). Estrogens stimulate the proliferation of lactotroph cells, PRL synthesis and reduce dopamine release (76, 77). That could explain the higher incidence of PRLomas in women. There is also a report of PRLoma development in a transgender male under high doses of estrogen therapy (78). Likewise, the Dutch transgender registry shows that the incidence of PRLomas is higher in transgender women compared with cisgender women (79); however, another study does not support such observation (80). Moreover, no association between estrogen exposure in women taking oral contraceptive pills and a higher incidence of PRLomas were identified (81, 82). Also, no significant correlation was found between estrogen concentration, nor with tumor size or Ki-67 expression (68).

In men, PRL-secreting PAs express lower levels of ERα than in women (72, 73). Delgrange et al. have demonstrated that low expression of ER correlates with clinical (tumor size, invasion and progression, DA resistance and worse surgical outcome) and pathological (higher Ki-67 index, mitotic count and p53 expression) factors of worse prognosis (72). Mahboobifard et al. additionally studied a different expression pattern of ER variants (ERα66 and ERα36) in PRLomas. Invasive, recurrent and DA-resistant tumors presented low expression of ERα66 and ERα36; moreover, low ERα36 expression was associated with higher Ki-67. All aggressive macroPRLomas in males in the study showed low ERα66/low ERα36 phenotype (74). Therefore, the level and specific phenotype of ER expression may account for the observed higher risk of more aggressive PRLomas behavior in men and indicate tumor less differentiation.

Some studies also indicate progesterone involvement in PRLomas development (83–88). Animal models show that PRLomas have lower expression of progesterone receptors (PR) compared with normal lactotroph cells (86, 89), however, they present a significantly higher proportion of membrane PR (mPR) versus nuclear (nPR) (87). Moreover, in male mice pituitaries, mPR represents around 80% of PR expression, whereas in females, around 45% (87). Progesterone via mPR on lactotroph cells inhibits the secretion of PRL and indirectly enhances dopaminergic activity (88). The exact effect of the sex difference in nPR and mPR expression in the pituitary and its involvement in PRLomas pathogenesis is not fully understood. It is speculated that in males, the higher pituitary mPR expression could be protective from PRLomas development (90).

### 3.3 Growth factors

Among the growth factors involved in the regulation of the pituitary function, particular attention to PRLomas development is paid to the transforming growth factor-beta1 (TGFβ1) due to its involvement in lactotroph physiology. TGFβ1 inhibits the proliferation of lactotroph cells and PRL release by influencing dopamine and estradiol’s effect on lactotroph cells (91–93). According to some data, PRL-secreting PAs present lower TGFβ-system activity than normal lactotroph cells (91, 92, 94). However, contrary data show that DA-resistant PRLomas with high fibrosis present higher levels of TGF-β1 system signaling (95, 96). Male mice presented higher pituitary TGFβ1 system activity in animal models than females (97). There is a suggestion that sex-related TGFβ1 expression in the pituitary is associated with sex-related differences in PRLomas pathogenesis and could protect males from excessive lactotroph proliferation, PRL secretion and PRLoma development (98).

Another growth factor, vascular endothelial growth factor (VEGF), has been reported to be overexpressed in PRL-secreting PAs (99), and that overexpression is higher in men than in women (73). Since the VEGF pathway promotes tumor angiogenesis and invasion (100), larger and more invasive PRLomas, are better vascularized than noninvasive tumors (98), which could explain why PRLomas in males are, in general, more well-vascularized when compared to females.

A recently published animal model study also showed that the kinin system might be involved in the development of PRLomas. The main bradykinin receptor (BR) in lactotrophs is the B2 subtype (B2R), and its expression is higher in males than in females. B2R activation stimulated PRL secretion in males and inhibited PRL secretion in females, and stimulation of dopaminergic activity had an inhibitory effect on B2R gene expression in females but not in males. Increased B2R concentration was observed in mutated mice pituitaries developing PRLomas compared with wild types (101).

### 3.4 Molecular pathways and genetic alterations

As mentioned previously, PRL-secreting adenomas can arise due to germline mutations in the case of familial genetic syndromes (66, 67). Compared with other PAs, the genetic causes of sporadic PRLomas tumorigenesis remain unknown. However, Lil et al. identified a hotspot somatic gain-of-function mutation in splicing factor 3 subunit B1 gene (*SF3B1*) in up to 19.8% of PRLomas that appears to be a specific genetic alteration for PRL-secreting adenomas. The *SF3B1* mutation leads to aberrant alternative splicing of estrogen-related receptor gamma (ESRRG), resulting in ESRRG’s stronger affinity for pituitary-specific positive transcription factor 1 and leading to lactotroph cells proliferation and PRL hypersecretion. *SF3B1* mutants demonstrate higher PRL levels and poorer prognosis when compared with the wild-type group. The mutation frequency in the studied group was significantly higher among men than women (24.34% and 10.67%, respectively) (102). In the recently published multicenter study in Europe by Simon et al., *SF3BP1* likely pathogenic variants were identified only in 2.5% (7 out of 282) PRL-secreting PAs. *SF3BP1* variants were associated with more aggressive tumor behavior and worse clinical outcome, and 50% of PRLomas harboring that pathogenic variant were metastatic (103).

Moreover, additional genes were shown to be overexpressed in PRLomas. Approximately 18% of the genes differentially expressed between male and female PRLomas have been reported to be involved in the estrogen signaling pathway. Werinecki et al. indicate three of them located on the X chromosome (*CTAG2*, *FGF13*, and *VEGF-D*) as potential candidates in sex-related dysregulation in PRL-secreting PAs (73).

Several chromosomal abnormalities (mainly of chromosomes 1 and 11) have been identified in PRLomas and were more numerous in more aggressive tumors. One of them (gain of chromosome 19p) was found to be specific for aggressive tumors in men. Four genes (*CRB3*, *FAM138F*, *MATK*, and *STAP2*) located on gained chromosome 19 region were found to be upregulated in PRL-secreting PAs in men, and two of them (*MATK* and *STAP2*) are involved in the estrogen signaling pathway. It is speculated that in males low level of ER could increase *STAP2* expression and be involved in the aggressiveness of male PRLomas (73). These findings support the speculated role of the estrogen signaling pathway in observed sex-related dimorphism of PRLomas and the more aggressive character of PRL-secreting PAs in men.

## 4 Diagnosis

### 4.1 Biochemical diagnosis

Many factors, involving physiological ones, can result in hyperprolactinemia (104–106). In the course of diagnostics, it is recommended to exclude renal failure, hypothyroidism or the usage of drugs affecting hypothalamic-pituitary regulation of PRL secretion (39, 104–108). In contrast to women, symptomatic hyperprolactinemia in men with normal pituitary images on MRI is rare (109). In the Colao et al. study, among 74 male patients with symptomatic hyperprolactinemia, none were diagnosed with a non-tumoral origin (50). Endocrine Society guidelines state that a single measurement of serum PRL concentrations permits the diagnosis of hyperprolactinemia (39). To include possible pulsatile PRL secretion, sampling at 15-20 minute intervals may be considered (39). Dynamic tests are not recommended (39). Patients with hyperprolactinemia but serum concentration of PRL less than five times the upper limit of normal should undergo PRL retesting (3). In patients with asymptomatic hyperprolactinemia, evaluation of macroprolactinemia should be considered (6, 39, 105, 106). Macroprolactinemia, however, is significantly less frequent in men than in women (6.28 and 15.03%, respectively) (110). Baseline PRL concentrations before treatment correlate with tumor size (5, 6, 14, 70, 111), and thus due to the larger diameter of tumors in men, PRL levels in this group of patients are significantly higher than in women (5, 14, 20, 31, 68). Discrepancy should trigger consideration of other possible causes of hyperprolactinemia (3). Low PRL concentration in relation to adenoma diameter may be a manifestation of low tumor differentiation (70) or the result of the hook effect, especially in tumors ≥ 3 cm with normal concentrations or mild hyperprolactinemia ≤ 200 ng/mL in laboratory findings (39, 106, 112). Although the hook effect in assays is rare (112), serial dilution of samples at a ratio of 1:100 is recommended to eliminate the test artefact (39). A value greater than 200 ng/mL usually suggests the presence of PRLoma, and those exceeding 500 ng/mL are characteristic for macroPRLoma (39). In patients with gPRLoma, PRL concentration generally is above 1000 ng/mL (106).

Due to the possibility of mixed secretion of GH and PRL, insulin-like growth factor-1 (IGF-1) assessment is recommended for patients with PRLomas at diagnosis, while its long-term monitoring is not recommended (113). Particular attention should be directed to patients who demonstrate a significant decrease in PRL levels with no corresponding decrease in tumor diameter (114).

### 4.2 Radiological diagnosis

Men tend to develop larger diameter tumors which, compared to women, are more likely to infiltrate the cavernous sinus (4, 7). GPRLomas, in addition to infiltration of the cavernous sinus, may invade the clivus, sphenoid, and ethmoid sinus, and thus, depending on the direction of invasion, may be connected with impaired hearing, unilateral hemiparesis, and temporal epilepsy (7). MRI is the dedicated examination for evaluating the pituitary gland and structures surrounding the sella turcica (115). To assess the cavernous sinus invasiveness in order to standardize radiological criteria, the Knosp criteria are used, which classify parasellar growth in five grades, also taking into account the internal carotid artery as a radiological landmark (116). Knosp criteria, along with early postoperative PRL levels, are clinically significant early predictors of remission in patients after transsphenoidal resection (117). While T2 intensity may correlate with the histologic subtype of somato- and corticotropic PAs, data on the use of T2 intensity in PRLomas are limited (115, 118); however, most PRLomas are hyperintense on T2-weighted images (118). The Kreutz et al. study revealed that PRLomas in women are high signal intensity (SI) (91%) tumors on T2-weighted images, with a small percentage of iso-SI (6%) and low-SI (3%) (4). In contrast, in men, the percentage of tumors with high-SI was 39%, and iso-SI and low-SI: 46% and 15%, respectively (4). The T2 signal heterogeneity of PRLomas may be associated with clinical features and be a predictor of response to DAs treatment (119). Heterogeneous T2 intensity signal PRLomas have a higher occurrence in men and reflects intratumoral necrosis and hemorrhage (4, 119). Hypointense PRLomas were correlated with resistance to DA treatment (118). Large-diameter pituitary adenomas, including PRLomas, may show degenerative features such as necrosis, calcification, hemorrhage or cyst formation. These attributes are more common in men (9).

Computer tomography (CT) also has its role in the diagnosis of patients with PRLomas, useful in visualizing calcifications or cellular floor bone defects (120). Radiological detection of calcifications on CT is useful in differentiating PAs from other perisellar lesions (121, 122). The most frequent lesions with calcifications in the sellar area are craniopharyngiomas (121), but they may also occur in PAs and Rathke’s cleft cysts (5). The incidence of radiologically confirmed calcifications in PA varies from 0.2 to 14.0% (121), but usually does not exceed 2% (122). However, a higher incidence has been reported after histopathological verification-pituitary calcifications have been described more frequently in cases of hormonally active PAs, especially those secreting PRL compared to other hormonally active ones (123). Calcifications in PAs may also be subdivided according to the histologic pattern – scattered psammoma bodies between the cells of the adenoma are a characteristic of calcifications found in PRLomas (122). Damage to the floor of the sellar is more common in macroPRLomas, and clinically may manifest as cerebrospinal fluid (CSF) leakage (124).

## 5 Clinical manifestations

Diagnosis of PRLoma among men is made later than in women (12–14, 19). While female patients seek medical advice earlier for menstrual disorders, galactorrhea or infertility, diagnosis in men may be delayed due to less awareness of symptoms or the non-specific, and sometimes shaming, nature of patients (9, 12–14, 19, 25, 26). In men with hyperprolactinemia, the diagnosis is determined at an older age, which also postpones the possibility of early sexual disorders detection (30). Delayed diagnosis alone impinges on the size of the tumors at the time of identification (30), but it is not an isolated factor affecting their size or revealing aggressive tumor features (6). This is also influenced by sex-related differences and receptor pathways (6), as described in previous sections of the review. The timing of tumor diagnosis also affects the chance of successful treatment, including surgical procedures (70). The clinical manifestation varies markedly in the male and female patients at the time of reporting (30). Elderly men with PRLoma might be symptom-free (18). In this subgroup of patients, the diagnosis may be made after imaging studies for other indications (18), and almost 10-20% of all diagnosed pituitary tumors may be detected accidentally (125). The symptoms of PRLomas can be classified as secondary to hyperprolactinemia or due to the tumor mass effect (107), in the case of larger tumors (12). Because of the observed greater diameter of the tumors, other early signs in men, including symptoms not directly related to increased PRL levels but to the mass effect, may occur - visual disturbances (9, 12–19, 21–23, 68) that appear much more frequently than in women (14), or headaches (12, 13, 15–17, 19). In large-diameter pituitary tumors, adenoma apoplexy may occur (6, 12). MacroPRLomas can infiltrate the cavernous sinuses, but cavernous sinus syndrome itself, with a violent onset and progressing with ocular paralysis or visual disturbances, is rare and usually associated with pituitary apoplexy (6).

Rare complication of treatment is CSF leakage (124), which usually may occur in gPRLomas with rapid reduction in tumor diameter due to DA treatment (120). Factors that increase the risk of CSF leakage are male gender and DA resistance. CSF leakage is frequent after surgery with transsphenoidal access for sellar lesions and anterior skull base lesions (124).

It was also found that in boys macroPRLomas are more likely to affect puberty than in girls (56). Despite similar PRL concentrations, menstrual disorders are the most common sign in women (7, 20, 50), whereas in men: erectile dysfunction and decreased libido (7, 9, 12, 13, 15, 17–23, 31, 107). In males, infertility is the less common reason for medical consultations than in women (30), and galactorrhea is among the rarely recognized symptoms (12, 31).

**Figure 2:**
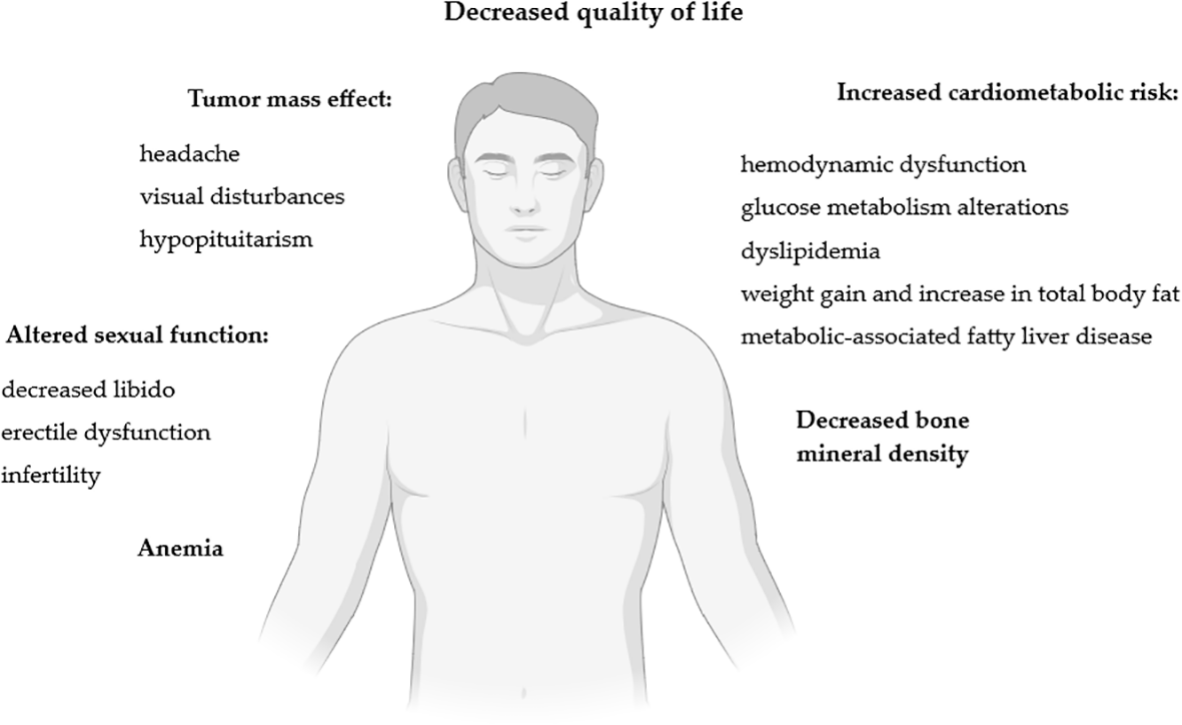
Impact of lactotroph tumors on men’s health. Figure created in BioRender.com.

The impact of lactotroph tumors on men’s health is summarized in **Figure 2**.

### 5.1 Quality of life

Patients with pituitary adenomas have been shown to demonstrate a lower quality of life (QoL) (126) with improvement for all hormonally active PAs once disease control is achieved, except for acromegaly (127). While dedicated scales, such as AcroQoL, are available for acromegaly, no intentional assessment questionnaires exist for PRLomas (128). QoL of patients with PRLoma is less impaired than in acromegaly or hypopituitarism, but patients reported symptoms, especially those related to sexual dysfunction, significantly impact QoL (128). Data assessing female patients with microPRLoma using the NHP, SP-36, MFI-20, and HADS scales are available (129), but information on the evaluation of men with PRLoma seems to be limited.

### 5.2 Hypopituitarism

In the Iglesias study, hypopituitarism at the time of PRLoma diagnosis was found in about 75% of patients and was more frequently diagnosed in patients with macro- than microPRLomas (15). In high-diameter pituitary adenomas, including gPRLomas, in addition to HH, clinical manifestations consist of effects on the somato- (39-83%), thyro- (18-41%) and corticotropic (12-67%) axis (111, 130–133), which most likely should be related to tumor diameter (12, 16). Nevertheless, the gonadotropic axis is most often disturbed, regardless of tumor diameter, mainly due to the negative effect of hyperprolactinemia itself on gonadotroph function (134). In fact, in men with macroPRLomas, at the time of the diagnosis, HH occurs in about 76-100% of them (135, 136). The PRL-to-testosterone ratio should be considered an effective independent indicator of MRI imaging pituitary abnormalities (137). Despite this, testosterone levels in men with PRLomas may be normal, and this fact should not exclude the diagnosis (12, 138). Testosterone levels typically increase after initiation of DA treatment (37, 139). However, in some men (11-73% depending on the study), HH persists even after hyperprolactinemia normalization (31, 131, 133–136, 140, 141). The proposed independent predictors of HH persistence in men PRLomas include higher baseline PRL levels (135, 136, 141, 142), larger tumor size (16, 135, 136, 141, 142), lower baseline testosterone levels (135, 140–142), hypopituitarism (140, 142), and visual field defect (140). It is postulated that persistent HH in males with PRLomas is mainly an effect of chronic hypothalamus functional modification caused by the inhibitory effect of PRL (136, 143), and, to a lesser extent, derives from direct structural damage of the pituitary (24).

Nevertheless, resolution of HH in patients with macroPRLomas is obtained in a significant proportion of cases upon DAs treatment and PRL normalization. Several studies have attempted to evaluate recovery of pituitary dysfunction, other than the gonadotroph axis, in patients with macroPRLomas and hypopituitarism; however, they are based on a small number of patients with different diagnostic criteria among series (111, 130, 133, 144, 145). The frequency of potential somato-, thyro- and corticotropic axes recovery is not clearly defined, as it differs significantly among the various studies. On the one hand, it is postulated that recovery of hypopituitarism, other than the gonadotroph axis, is relatively infrequent (133); on the other hand, there are reports of somato-, thyro- and corticotropic axes recovery in 62,5-67%, 45% and 60-67% of patients respectively (111, 130, 145), which may lead to contradictory conclusions. However, as the tumor size decreases, normal pituitary function might be expected to return and these patients therefore require frequent endocrine re-evaluation.

### 5.3 Anemia

In male patients with macroPRLoma, mild anemia exactly connected with hypogonadism may occur (12). In the Ellegala et al. study, men with PAs had decreased testosterone concentration and associated anemia, with improved hematocrit after testosterone replacement therapy (TRT) (146). In another study by Shimon et al., anemia was diagnosed in almost all subjects and improved in line with a reduction in PRL concentrations and an increase in testosterone levels (19). In the recently published study by Rudman et al. it has been shown that marked decrease in hemoglobin levels in men preceded PRLoma diagnosis by a median of 6.1 years, with a mean delay of 4.1 years between hemoglobin decrease and HH symptoms onset (147).

### 5.4 Impact on metabolism

Another important issue in men with PRLomas is their metabolic profile. Indeed, men with hypogonadism are at increased risk of developing metabolic syndrome (MS) (12, 28). However, regardless of HH, in men increased PRL concentrations itself appear to impair gluco-insulinemic profile and body composition, causing glucose intolerance, hyperinsulinemia, insulin resistance (148, 149), weight gain, and increase in total body fat percentage (especially in the arm and leg areas) (29, 37, 38). In addition, hyperprolactinemia influences appetite regulation and increases food intake, leading to weight increase, regardless of PRL metabolic effect (29, 150, 151). The Auriemma et al. study revealed that cabergoline (CAB) treatment in men with hyperprolactinemia improves their metabolic profile and reduces the incidence of MS. However, in the case of persistent HH, an improvement in MS usually requires and might be achieved by the implementation of testosterone treatment (152).

The direct relationship between hyperprolactinemia and BMI itself remains controversial. Potential mechanisms in which hyperprolactinemia may induce weight gain include decreased dopaminergic tone and leptin resistance (28, 153, 154). In a study by Posawetz et al., men and women with PRLoma had higher BMI than the control group (37), while in another by Dos Santos et al., BMI did not correlate positively with PRL concentration (28). The relation between leptin levels and hyperprolactinemia is inconclusive. In a study by Pala et al., patients with PRLomas revealed higher leptin levels compared to controls, which decreased after 6 months of CAB treatment (154). In contrast, a study conducted by Dos Santos et al., revealed no correlation between prolactin and leptin concentrations (28).

### 5.5 Cardiovascular risk

Men with PRLoma have an increased risk of cardiovascular disease (CVD), which was not found in the group of female patients with the same diagnosis (27). Indeed, patients with PRLomas are likely to present dyslipidemia and have increased total-cholesterol, LDL-cholesterol and triglycerides and decreased HDL-cholesterol levels (29, 155). An unfavorable lipid profile may further promote the development of hepatic steatosis (151). In the Andreggen et al. study, total cholesterol and LDL fraction levels decreased after initiation of CAB treatment in both male and female groups, however, the change was more prominent in males (139). Another study involving young patients with mild idiopathic hyperprolactinemia reported no effect on peripheral and central blood pressures and pulse wave (156). However, in a recent study by Jurek et al., male patients newly diagnosed with PRLoma presented subclinical hemodynamic dysfunction assed by impedance cardiography, despite normal blood pressure values (157). That leads to the conclusion that evaluating the long-term impact of hyperprolactinemia on CVD requires further research. Also of interest remains the statement that macroprolactinemia increases the cardiometabolic risk (CVD + type 2 diabetes) to a lesser extent than monomeric hyperprolactinemia (158). However, the Framingham Heart Study demonstrated that PRL is not associated with cardiovascular risk factors, and the evaluation of PRL concentrations does not provide a meaningful assessment of cardiometabolic risk (159).

### 5.6 Bone health

Increasing PRL concentrations of mild grade still stimulate bone remodeling, but with resorption predominating over bone formation (160). Significant hyperprolactinemia increases bone resorption, but inhibits bone formation - which impairs the trabecular structure of the bone (160). Patients with hyperprolactinemia have been found to have decreased osteocalcin, high levels of N-telopeptide (161) and an increased ratio of RANKL to osteoprotegerin (160, 162). Persistently elevated PRL levels and male sex are independent factors for decreased bone mineral density (BMD) (35, 107, 139, 163). The impairment of BMD and fracture prevalence correlate with hypogonadism’s duration and severity (12, 35, 36). While HH is known to have adverse effects on BMD (160), it has been shown that hyperprolactinemia can cause negative effects on the skeletal system regardless of testosterone concentrations (12, 35).

In a study led by Mazziotti et al., fractures occurred less frequently in patients treated with CAB than in untreated patients (35). In the Di Somma et al. study, after 18 months of DA treatment, despite successful therapy of hyperprolactinemia in men, BMD of the lumbar spine and femoral neck were unable to normalize (161). Similarly, in the Colao et al. study, bone mass in young patients did not return to normal even after two years of treatment with DA (36). The last two studies mentioned have some limitations due to the short follow-up period. However, they are worth using because most of the available data on the association of BMD during DA treatment concerns females, and those relating to males appear to be limited. In the Di Somma et al. study, however, BMD improvement was observed during treatment, suggesting that effective therapy of hyperprolactinemia may prevent the progression of osteopenia and osteoporosis (161).

### 5.7 Impact on fertility

Assessment of PRL levels should be remembered during the diagnosis of male infertility (164, 165). Considering spermatogenesis as a complex process dependent on endocrine and paracrine/autocrine testicular factors (166), it is important to present the role of PRL. The hypothalamic-pituitary-gonadal (HPG) axis is crucial for semen production and maintenance of sexual function in men (32). Hyperprolactinemia has been shown to directly affect spermatogenesis by inhibiting the pulsatile secretion of GnRH (30, 32, 33, 167) and thus reduces the pulsatile secretion of the major regulators of spermatogenesis: FSH, LH, and testosterone, affecting semen parameters (32–34). Hyperprolactinemia may lead to oligozoospermia, decreased motility and abnormal sperm morphology (13, 30, 31). PRL also directly affects the testes, inhibiting gonadotropin production (32) by affecting receptors for PRL in Sertoli and Leydig cells (34, 168).

A number of studies from the past few years have suggested a major role for PRL in the context of male infertility, but in reviewing existing data involving men undergoing endocrine evaluation for abnormalities in semen parameters, hyperprolactinemia was found in widely varying percentages: from 3.6% for secondary and 8.07% for primary infertility (169), through 12.2% (170), 12.5% (171) to as high as 16.7% (164). These results show very high variability due to the diversity of the study population, the method of measurement or the criteria for diagnosing hyperprolactinemia (164). DA treatment leads to clinically significant improvement in semen parameters (135, 167) and sexual function (30, 172) while reducing tumor size and returning gonadal function in patients with symptomatic PRL-secreting adenomas (39).

On the other hand, it is also worth mentioning the Corona et al. study, which examined the association of hypoPRLemia with sexual dysfunction and showed that a higher risk of erectile dysfunction and premature ejaculation was found in patients with lower PRL levels (173). An older study by Gonzales et al. demonstrated an association between hypoPRLemia and decreased sperm motility (174).

### 5.8 Impact on prostate

PRL is an important component in regulating of citrate production by prostate epithelial cells, thus the prostate’s main function (175). In addition, it inhibits apoptosis induced by androgen deprivation treatment and promotes the proliferation of epithelial cells, normal as well as neoplastic prostate (176). In animal studies, it has been shown that hyperprolactinemia can induce changes specific to a particular lobe of the prostate gland and so, for example, moderate intensity hyperprolactinemia caused hypertrophy in the ventral lobe and hyperplasia in the dorsal lobe (177). Based on studies in rodents, the involvement of PRL in benign prostate diseases [benign prostate hyperplasia (BPH), inflammation] has also been suggested, but there are currently no data on its role in the pathogenesis of these conditions in humans (178). It was not shown that PRL receptor expression was increased in BPH compared to normal prostate cells (178). Likewise, circulating PRL levels were not correlated with BPH (178). In contrast, Colao et al. showed no change in prostate structure in patients with hyperprolactinemia compared to controls, most likely because hyperprolactinemia is accompanied by low levels of testosterone and dihydrotestosterone (179). The PRL receptor is not overexpressed in human prostate cancers (180). The role in carcinogenesis should be attributed to local rather than circulating PRL (176). The collected data suggest that PRL, including that produced locally in the prostate gland, should be considered a promoter of benign and malignant prostate tumors, and the autocrine and paracrine mechanism itself should be considered a new therapeutic approach (178, 180). However, it is worth mentioning a case reported by Costello, in which inhibition of pituitary PRL production with CAB was an effective treatment in a patient with advanced prostate cancer (181).

## 6 Treatment

### 6.1 The aim of the treatment

The objectives of treatment of PRLomas in men consist in decreasing PRL levels, reversal of clinical signs, tumor shrinkage, restoring gonadal function and other pituitary hormone deficiencies, and preventing tumor recurrence or progression. The resolution of hypogonadism, symptoms control and prevention of hyperprolactinemia complications significantly influence the decision of whether to initiate treatment. Therefore, asymptomatic patients diagnosed with microPRLoma do not mandatorily require treatment (182). However, considering that the majority of PRLomas in men are macroadenomas with high serum PRL concentration, they ought to be treated in most cases (39). Also, since males more frequently present with more aggressive and invasive tumors, treatment of PRLomas in men could be challenging.

### 6.2 Pharmacological treatment with dopamine agonists

The treatment of PRLomas is unique amongst the PAs, since they are the only ones in which the first-line approach is a medical intervention with DA rather than surgery, regardless of their size, in both females and males. Treatment with DA should be started with a low dose, and titrated according to PRL levels, response and reduction in tumor size (39). Patients with macroPRLomas will require high-dose therapy and rapid dose escalation (183). However, a comparative prospective randomized study found that the time needed to normalize PRL levels and tumor shrinkage by 50% was comparable with intensive DA treatment to standard dosage schedule (184).

Wang et al. evaluated 8 randomized and 178 nonrandomized studies, including more than 3000 patients (185). DA, compared with no treatment, significantly reduced PRL levels and the risk of persistent hyperprolactinemia. PRL normalization was obtained in approximately 70% of patients, whereas tumor reduction was observed in approximately 60% (185).

The effectiveness of DA treatment has been reported to be comparable for the male-only population to the numbers achieved by the population of both sexes and the normalization of PRL levels in monotherapy was found in approximately 70% of patients (15). In clinical practice, the most used DA are CAB, bromocriptine (BRC), and quinagolide. Due to its efficacy, long-acting effect, and relatively uncommon adverse events, CAB is usually used as a first-line treatment (186). BRC is considered to be less effective than CAB in reducing the risk of persistent hyperprolactinemia but is similarly effective in reducing the PRL levels (51, 187).

Lin et al. reviewed publications (2000–2018), which included 1,362 patients, and found that treatment with CAB normalized serum PRL levels in 79.7% of them. That was achieved in 84% of patients with microPRLomas, and in almost 77% with macroPRLoma. Tumor reduction was reported in around 74% of patients and side effects were reported only in 5.1% of them (188). Other studies in a large populations have shown similar efficacy of CAB treatment (50, 189). Colao et al. showed that there are no significant differences between the sexes regarding the response to CAB therapy. Similarly, studies evaluating the efficacy of DA treatment of PRL-secreting PAs in the male population only, have shown similar effects to that described above in the general population, regardless of initial tumor size (31, 111). Additionally, it has been reported that normalization of PRL is similar in men with micro- and macroPRLomas (83% vs 79%) after DA monotherapy (134).

The clinical response is highly variable. In some females, complete PRL normalization is necessary before ovulatory menstruation can resume, but in other cases, clinical improvement may be seen despite persistently abnormal PRL levels. In contrast, testosterone levels in males often return to normal only after the normalization of PRL levels, which may take several weeks. Therefore, the dosage of CAB should be customized for each patient (40, 51).

### 6.3 Adverse effects of dopamine agonists

Although DAs benefits outweight their shortcomings, it should be mentioned that the use of these agents is not free of side effects, some severe enough to justify discontinuation of this medical therapy favouring others, especially surgical intervention.

DAs’ most reported side effects include nausea, vomiting, headache, and hypotension, with rare side effects of rhinorrhea and hypotonia (185). Less known but causing much concern in men treated with DAs, in whom may be reports of a much higher incidence of impulse control disorders, including gambling, compulsive shopping, banging eating, and hypersexuality (known as “dopa-testotoxicosis”), which is more likely to be developed by male patients with previous history of such behaviors (190–192). One possible reason for this phenomenon is that increased testosterone levels may cause it during a prolonged hypogonadal condition in combination with the stimulation of the reward system by DA therapy (193). The patients should be warned about the risk of such behavior; if it occurs, a drug dose reduction may be needed.

Another adverse effect that raises a lot of uncertainty is the potential cardiac valve involvement. In 2008, Colao. et al. assumed that moderate tricuspid regurgitation might be more frequent in patients taking CAB, and it could correlate with higher doses of CAB (194). It caused much concern, especially for the male population, which, as mentioned earlier, is more likely to require larger doses of CAB. Following that, several research studies, including a recent population-based cohort study, have addressed this issue, and in most cases, no connection between DAs and heart valve disease was found (195–198).

### 6.4 Dopamine agonists withdrawal

While medical therapy is generally considered a lifelong therapy for most patients, it has been shown that the complete withdrawal of DA may be successful under well-defined conditions. Most studies on DA withdrawal indicate that the most effective predictor of long-term remission is the absence of a tumor on MRI before withdrawal (6). According to Endocrine Society Guidelines, dosage reduction or discontinuation of therapy should be considered when normalization of serum PRL levels and disappearance of pituitary tumors are achieved after two or more years of DA treatment (39). Patients with higher PRL levels at diagnosis, large and invasive tumors, and those who have not responded well in the initial months of therapy, which is more often in men, have lower chances of achieving the withdrawal criteria (199).

### 6.5 Resistance to dopamine agonists

In most patients, implementation of standard DA therapy allows for PRL normalization and adequate tumor mass reduction. The failure to achieve normal PRL or lack of relevant tumor shrinkage (≥ 30% in maximum diameter or ≥ 50% in volume) when treated with standard DA doses (10 mg per day of BRC or 2 mg per day of CAB) at least 6 months is defined as DA resistance (3, 200). The overall prevalence of DA resistance is 10% for CAB and 20-30% for BRC. Patients with BRC-resistant PRLomas should be switched to CAB treatment, as CAB is more effective in PRL suppression and tumor control (3, 201). In the case of CAB-resistant PRLomas, it is advised to increase the dose to the maximum tolerated (3, 202).

Male sex and cavernous sinus invasion are the two main clinical factors significantly and independently associated with resistance to the treatment (10, 11). The molecular mechanism that leads to DA resistance is uncertain. In *in vitro* (203) and animal model studies (89) aggressive and resistant PRLomas present with lower expression of dopamine D2 receptor (D2R). The D2R exists as one of the two alternatively spliced isoforms, short and long; the reduced expression of long D2R isoform has been correlated with DA-resistance (204). Studies have also shown that filamin-A is crucial to maintaining proper D2R expression and signaling, and the impaired response to DA in resistant PRLomas may be related with low filamin-A expression (205). D2R polymorphism has also been evaluated, and in a study by Filopanti et al., the NcoI-T allele was associated with CAB resistance. No association between D2R polymorphisms and sex was observed in the analyzed group (206). However, another study found no correlation between D2R polymorphism and CAB responsiveness in patients with PRLomas (207).

In the recent study by De Castro Moreira et al., it has been suggested that different variants of PRL receptors may be associated with tumorigenesis of PRLomas and CAB resistance; patients with specific PRL receptors variants presented higher serum PRL levels, larger tumor size at diagnosis, higher frequency of CAB resistance with male predominance (208).

In the case of aggressive and refractory PRLomas and PRL-secreting PC (both more common in males), the use of temozolomide (TMZ), an oral alkylating chemotherapeutic, might be considered (65, 209). In a cohort study by the European Society of Endocrinology, aggressive PRLomas and PRL-secreting PC comprise 20.0% (25/125) and 37.5% (15/40) of aggressive PA and PC, respectively. TMZ led to complete (5.0%) or partial (45.0%) tumor regression in approximately 50% of lactotroph tumors and stable disease in 26.0%; further tumor progression was observed in 24.0% of patients upon TMZ treatment (65). In a subsequent European Society of Endocrinology survey of 171 patients with aggressive PA and PC, lactotroph tumors comprise 54 cases (38 [31.4%] aggressive PRLomas and 16 [32.0%] PC). TMZ treatment in 156/171 patients resulted in complete response in 9.6%, partial response in 30.1%, stable disease in 28.1%, and progressive disease in 32.2% of the patients (64). Longer duration (>6 months) of TMZ treatment, its early use and its combination with radiation therapy might improve outcomes (3). However, after a limited response period, many patients escape from the beneficial effects of TMZ. Immunohistochemical evaluation of O6-methylguanine-DNA methyltransferase (MGMT) might assist in treatment decisions, as patients with low MGMT expression typically show a favorable response to TMZ treatment (210–212). However, low or absent MGMT may not always be a predictor of TMZ response – it has been shown that approximately 35% of aggressive PRLomas or PRL-secreting PC that progressed despite TMZ therapy presented with absent MGMT expression (213).

Therefore, it is necessary to search for new therapeutic possibilities. Data concerning the use of somatostatin analogues in the treatment of PRLomas is limited. However, there are documented cases of aggressive and DA-resistant PRLomas successfully treated with pasireotide, with PRL normalization and tumor size reduction (214, 215). Other options that have been studied in patients with aggressive PRLomas include targeted oncological agents, such as everolimus (216, 217), bevacizumab (64, 218, 219), lapatinib (220, 221) and immune-checkpoint inhibitors (ipilimumab and nivolumab) (218, 219, 222, 223). However, given limited and conflicting data, there is uncertainty about their effectiveness in resistant PRLomas.

As mentioned earlier, specific expression of ER plays a role in the pathogenesis of PRLomas and could be used as a predictor for tumor aggressiveness and response to therapy. Additionally, estrogen stimulation increases PRL synthesis; lowering estrogen levels might improve HH, decrease estrogen-stimulated PRL secretion, and increase DA sensitivity in refractory PRLomas. Therefore, the possibility of using ER modulators and aromatase inhibitors in managing DA-resistant PRLomas is postulated. In men with DA-resistant prolactinomas and persistent HH, the aromatase inhibitors (anastrozole and letrozole), when administered combined with high-dose CAB, resulted in a significant decrease in PRL concentration and tumor shrinkage (224, 225). In the recent systematic review assessing effect of tamoxifen in DA-resistant PRLomas, 20/22 patients (90.9%) responded positively to the use of tamoxifen with a mean reduction in PRL levels of 57.4%. 10 patients (45.5%) showed normalization of PRL post-tamoxifen administration. Combination therapy with DA and tamoxifen increased DA sensitivity and had a clinically significant inhibitory effect on PRL secretion. However, no male patients were included in this study (226).

An additional recognized factor of unfavorable response to classic pharmacological treatment is *AIP* or *MEN-1* germline mutated status. In the case of *AIP*-related PRLomas, most of these tumors occur in men, as opposed to sporadic PRLomas (227, 228). They occur at a significantly younger age, being larger and more invasive with poorer response to DAs than non-*AIP*-related adenomas (227–229). In MEN1 syndrome, PRLomas are the most common pituitary-related manifestation (228, 230). Although they are approximately 2.5 times more frequent in females, similar to *AIP*-associated cases, they pose a greater therapeutic challenge in males (227). PRLoma in a pediatric patient should prompt genetic testing for *AIP* and *MEN1* mutations (3, 231). Similarly, PRLomas in 3PA syndrome more frequently present as macroadenomas with aggressive behavior and resistance to standard treatment (228).

### 6.6 Testosterone replacement therapy

As mentioned previously, despite PRL normalization, HH may persist in up to 73% of men (31, 131, 133–136, 140, 141). Higher baseline PRL concentrations (135, 136, 141, 142), larger tumor size (16, 135, 136, 141, 142), lower baseline testosterone levels (135, 140–142), pituitary hormone deficiency (140, 142), and visual field defect (140) are considered as independent factors of its persistence. After observation in many studies, prolonging the follow-up period increases the percentage of men obtaining eugonadism (136, 140, 142). Therefore, before initiation of TRT, it is important to consider the possible presence of risk factors of persistent HH and the time since normoprolactinemia was achieved, usually starting after 3-6 months since then (12). There is a possibility that testosterone aromatization into estrogen could promote lactotroph cell hyperplasia and proliferation, leading to the development of DA resistance and tumor growth (232), thus treatment should be started carefully, preferably with short-acting preparations (gel applicants or injectable) (3). The appropriate time to include TRT has not yet been determined and requires further studies. It is suggested that treatment with HH should be initiated if testosterone levels have not normalized after six months of using DA (3, 111). However, some studies show that the recovery of the gonadotropic axis may occur later, even up to 48 months after the DA treatment initiation (133, 142). Therefore, periodic assessment of testosterone levels is essential to avoid unnecessary replacement therapy.

In men wishing to preserve fertility, TRT is contraindicated, gonadotrophin treatment might be considered (3). In case of persistent HH under DAs therapy, clomiphene citrate (selective ER modulator that antagonizes estrogen action on the hypothalamus) is a possible strategy for gonadotrophic axis recovery: clomiphene increases testosterone concentration and improves sperm motility independently of PRL normalization (233). Data on this approach are limited, however, given the recent information on the role of ER in the pathogenesis of PRLomas in men, the use of selective ER modulators in PRLomas management should be reconsidered.

### 6.7 Radiotherapy

The use of radiotherapy in patients with PRLomas is extremely uncommon due to the PRLomas’ good response rates to medical therapy. It is saved for tumors resistant to standard therapy, which are extremely aggressive, or malignant. Since we know that these types of tumors are more common in men, one might expect that radiation therapy for the treatment of PRLomas would be used more often in that population.

Radiotherapy techniques used to treat refractory PRLomas include external beam radiotherapy and stereotactic radiosurgery. While both have advantages and limitations, selecting a candidate for each method is important. One factor determining the best radiation strategy is the size of the tumor. Unless the tumor is larger than 3–4 cm or is within 3 mm of the optic nerves, chiasm, or tracts, radiosurgery is the recommended course of treatment (43).

Data from a single center in China with a long-term follow-up (average of 109.3 months) that included 24 patients demonstrated normalization of PRL levels using Gamma Knife Radiosurgery (GKRS) in 66.7% of cases, of which 41.7% occurred without and 25% with further DA therapy. All the patients showed tumor mass control (44). In a different study, 28 individuals (15 men) were monitored for a median of 140 months. 82.1% of patients, 46.4% with and 35.7% without adjuvant DA achieved PRL normalization. None of the patients had tumor growth (41). There were no sex differences described in these studies. GKRS may be a viable and safe therapeutic alternative for patients with PRLomas who are either intolerant or resistant to DAs, thus males are more likely to be eligible for such treatment.

Some retrospective investigations indicates that stopping DA treatment temporarily (2-4 weeks) before and during radiotherapy might increase the effectiveness of the radiation (234). However, this has not been confirmed, and it may be dangerous in cases of aggressive tumors, so each case should be considered individually (235).

A combination of radiotherapy with TMZ might be a preferred treatment option in aggressive PRLomas, as it shows better tumour control than either TMZ or radiotherapy alone (236).

Major side effects of radiotherapy include hypopituitarism, optic nerve damage, secondary brain malignancies, neurologic deficits, and higher risks of cerebrovascular accidents. Compared to conventional radiotherapy, stereotactic radiotherapy appears to have lower complication rates (42).

### 6.8 Surgery

Surgical treatment in PRLomas is typically considered only in rare cases of DAs resistance or extremely poor tolerance. It might be considered as the first-line therapy in pituitary tumor apoplexy, acute and progressive vision loss, spontaneous cerebrospinal fluid leak, and cranial hypertension (6, 237). Patients also might refuse medical therapy and opt for surgery as a first-line treatment. However, in the recent years a question of the place of surgery in the treatment of PRLomas has been raised (238–240).

The size of the tumor affects the surgical outcome. Chanson and Maiter, in a review that analyzed results from 12 series including a total of 1583 patients (741 with a micro- and 842 with a macroPRLoma), estimated that rates of remission (having PRL levels normalized within 1-12 weeks following surgery) were 81% for PRL-secreting microadenomas and 41% for macroadenomas. Depending on the surgeon’s experience and the chosen indications, surgical success rates varied greatly across the series, ranging from 60% to 93% for microadenomas and from 10% to 74% for macroadenomas (241). Considering that the majority of PRLomas in men are macroadenomas, it could be speculated that the overall effectiveness of tumor resection in men is lower than in women. Indeed, male sex and higher preoperative PRL levels has been shown as independent factors predicting persistent disease (242). In a retrospective series analysis, Yoo et al. showed that compared to women, men were much less likely to undergo complete gross resection during surgery and to see a normalization of the PRL levels following surgery (7). On the other hand, because male patients typically present with more aggressive tumors and increased likelihood of medical treatment resistance, male sex could be considered a factor favoring earlier decision of implicating surgery (243). In the recently published Pituitary Society international Consensus Statement on diagnosis and management of PRL-secreting pituitary adenomas, surgical resection of microPRLomas and macroPRLomas (Knosp grade 0 and 1) by an experienced neurosurgeon should be discussed as a first-line treatment option. Such an approach offers a high chance of cure, is cost-effective and avoids long-term DAs treatment (3).

The possibility of hyperprolactinemia recurrence following initial remission limits the effectiveness of PRLoma neurosurgery. Invasiveness of the tumor and early postoperative serum PRL concentrations are considered to be predictors of early remission following transsphenoidal PRLoma resection (117). Pituitary surgical experience is another important factor of the PRLomas resection outcome (240). After a median follow-up of five years, the overall recurrence rate is about 18% (241). However, in case of persistent hyperprolactinemia, there is a considerable decrease in PRL levels postoperatively compared to preoperatively under a lower DA dose in patients with preoperative DA resistance treated again after surgery; up to half of them normalize the PRL level (244).

In general, depending on the tumor size, invasiveness, PRL levels, and indications, surgical treatment for men presents with wide success rate, ranging in different series from 0 to 83% (22, 68, 163, 242, 243, 245). In conclusion, surgery is a viable alternative to standard medical therapy (especially for individuals who are intolerant or resistant to it). In the further perspective, it could be speculated, that the role of surgery in PRLomas management will be less restricted.

### 6.9 Treatment of gPRLomas

DAs (primarily CAB) are proven to be an effective and well-tolerated first line of therapy in men with gPRLomas. Regardless of their size, in the majority of cases, reduction or normalization in serum PRL and significant tumor shrinkage are achieved. DA therapy is considered superior even to surgical and radiotherapy treatment (16, 246). Nevertheless, there are reported cases where there was necessary to use both these therapies alongside others, such as TMZ and somatostatin receptor ligands (247, 248). Interestingly, in the recently published systematic review by Lisa et al., men with gPRLomas had a better response to DA treatment compared to women – PRL normalization was achieved in 55% and significant tumor shrinkage in 91% of men, whereas in 38% and 76% in women, respectively (59).

### 6.10 Conclusions

Although PRL-secreting PAs having a higher prevalence in women, those in males often pose challenges in clinical practice and have a multidirectional impact on male health. Regardless of symptoms directly related to the hyperprolactinemia, men tend to be diagnosed more frequently due to features of tumor mass effect rather than HH. Compared to women, PRLomas in men typically have a higher diameter, more invasive character, and more frequently present a lack of response to typical management, thus male patients might require a multifaceted therapy and individualized approach. Recent studies revealed that those mentioned sex-related clinical differences are linked with pathological distinctions in both sexes, including estrogen and progesterone signaling pathways, growth factors expression and specific molecular alterations. Current study methods provide an increasing amount of data on the genetics of sporadic PAs, as seen in recent reports on the increasingly better-understood genetics of corticotropic PAs. However, the data on the genetic basis in sporadic PRLomas are limited and require further investigation. A thorough knowledge of the molecular background of sporadic PRLomas tumorigenesis could explain the occurrence of male-specific characteristics. Finally, further research and exploration of new therapeutical options will allow more male patients with PRL-secreting PAs to achieve therapeutic goals.

## Author contributions

LD: Conceptualization, Formal Analysis, Writing – original draft, Writing – review & editing. JS: Conceptualization, Formal Analysis, Writing – original draft. ZZ: Conceptualization, Formal Analysis, Writing – original draft. WR: Writing – review & editing, Supervision PW: Writing – review & editing, Supervision.
